# Morgan County Medical Society

**Published:** 1868-07

**Authors:** C. T. Wilbur

**Affiliations:** Sec’y.


### MORGAN COUNTY MEDICAL SOCIETY.

The Society met in Firemen's Hall, Jacksonville, on Thursday, May 14th, at half-past eleven o'clock A.M. Upon being called to order by the President, Dr. Henry Jones, the proceedings of the last regular meeting were read and approved.

The committee on examinations having reported favorably upon the nominations of Dr. C. G. Jones, Dr. G. H. Sandford, and Dr. Welch, they were unanimously elected members of the Society.

This being the annual meeting the Secretary's report for the year was then read, and a resolution of thanks to Dr. Wilbur was passed.

The Treasurer, Dr. Craig, having been away from home, was unable to present his annual report, but promised to hand it in at the next meeting. A resolution of thanks to Dr. Craig for the faithful performance of official duties was then unanimously passed by the Society.

Nominations for delegates to the State Medical Convention to be held in Quincy, Illinois, on the 19th of May, were then made, and Drs. Fisher, Lucas, Edgar, Wilbur, De Leuw, and Johnson were elected.

Drs. Prince, H. Jones, W. S. Edgar, Bibb, and Fisher were then elected as the examining Committee of the Society for the ensuing year.

The Society then proceeded to elect its officers for the ensuing year with the following result:

For President—Dr. David Prince.

For Vice-President—Dr. J. G. Cox.

For Secretary—Dr. C. T. Wilbur.

For Treasurer—Dr. G. R. Bibb.

Dr. Prince was then conducted to the chair, when he made a few introductory remarks, followed by Dr. Henry Jones with a valedictory.

On motion Dr. Jones was tendered a vote of thanks by the Society, by a rising vote.

The following motion was then passed:

"That the corporate members of the Society present be assessed the sum of one dollar and fifty cents for the expense incurred in the Society dinner, and if the amount raised by said assessment exceed the expense thus incurred, the ballance shall remain in the hands of the Treasurer, subject to the order of the Finance Committee."

Dr. W. S. Edgar then read a draft of the proposed law for the "better regulation of the practice of medicine in the State of Illinois," and requested the opinion of the Society upon its practicability and main points.

Dr. Prince said, "The plan proposed seemed to him to be the only feasible plan. The prevalent feeling in the community seemed to be 'free medicine and no sects in medicine.' Public sentiment seemed to demand this—still the people need protection. Legislation is required to protect the public against ignorant pretenders. I should be glad if this plan should go to the State Medical Convention with the endorsement of the Morgan County Medical Society."

Dr. Samuel Adams then said, "I believe the popular sentiment is 'free medicine.' I think there is wisdom in Dr. Prince's suggestions, and the law should be shaped to avoid a collision with the sentiment of free exercise of each ones talent.

"I doubt the expediency of fine for prescription of remedies, for I think the forfeiture of remuneration would be a sufficient specification of penalty.

"Law cannot do much towards improving the practice of medicine. It must be done by the association, and proper united effort of educated men. I am not very hopeful of advantages to be derived from legislation.

"I would suggest that the evil lies partly where this proposition seems to place it, in the medical schools. They put through as many students as they can, and often when they are not properly qualified, in order to obtain large classes, and consequently considerable fees.

"This evil can only be remedied by properly endowed professorships—that will entirely remove the profession from the temptation of largess by reason of a large number of fees—or "no graduate, no fee." This law will have some influence upon these institutions by stimulating them to insist upon well qualified graduates. I am inclined to give this law my approval without being very hopeful of the success of such legislation.

Dr. Reed remarked, "I have not much hope as to the result of legislation. More depends upon the united action of men of education in the profession."

Dr. Fisher remarked, "I should hope a good deal from the passage of this law. It will serve to protect the community. When I was a student I happened to be in Paris in this State, when I had a conversation with a practicing physician, who advised me to go at once into the practice of the profession, and not spend further time or money in trying to get a diploma, for, he said, there was no particular advantage in the community for possession of the certificates of a regular medical education, and he consequently considered it time and money thrown away. I trust the passage of this law will ameliorate this condition of things.

Dr. Henry Jones remarked, "I have no doubt as to the expediency of making the effort. If we go before the community as trying to help them, they will surely consider us as acting selfishly. I am doubtful of results, but I should not hesitate to attempt to pass this law. I believe with Dr. Adams, that the elevation of our profession is the most probable means of doing good in this direction."

Dr. Craig remarked, "I have some hope that the bill may go through from its evident design. It protects the people from the demands of quacks. The Legislature will not dare to refuse to protect the people by placing safeguards around their pockets."

Dr. Henry Jones suggested "that this law unfortunately would not reach the most dangerous class of practitioners. Educated men who, with some ridiculous crotchet, would always practice upon the ignorance and credulity of the people of the community."

The motion was made and passed, that the plan of a law, as presented by Dr. W. S. Edgar, has the approval and hearty endorsement of the Morgan County Medical Society.

At 1.40 o'clock, the Society adjourned to meet at Dunlap House, for dinner, at 2 o'clock P.M.

C. T. WILBUR, M.D., *Sec'y.*

